# Cost-effectiveness of family psychoeducation to prevent relapse in major depression: Results from a randomized controlled trial

**DOI:** 10.1186/1471-244X-12-40

**Published:** 2012-05-14

**Authors:** Shinji Shimodera, Toshi A Furukawa, Yoshio Mino, Kae Shimazu, Atsushi Nishida, Shimpei Inoue

**Affiliations:** 1 Department of Neuropsychiatry, Kochi Medical School, Kohatsu, Okoh-cho, Kochi, 783-8505, Japan; 2 Department of Health Promotion and Human Behavior, Kyoto University Graduate School of Medical Sciences / School of Public Health, Yoshida Konoe-cho, Sakyo-ku, Kyoto, 606-8501, Japan; 3 Mino Clinic, Shimoishi, Kita-ku, Okayama, 700-0907, Japan; 4 Department of Psychiatry & Behavioral Science, Tokyo Metropolitan Institute of Medical Science, Kamikitazawa, Setagaya-ku, Tokyo, 156-8506, Japan; 5 Department of Health Promotion and Human Behavior, Kyoto University Graduate School of Medical Sciences / School of Public Health, Kyoto, Japan

## Abstract

**Background:**

Family psychoeducation is a relatively simple and straightforward intervention whose prophylactic effectiveness and cost-effectiveness is well-established for schizophrenia. We have recently demonstrated its effectiveness for unipolar depression**,** but its cost-effectiveness has never been examined. We hereby report a cost-effectiveness analysis alongside a randomized controlled trial in order to assess its cost-effectiveness for preventing relapse/recurrence in depression.

**Methods:**

Fifty-seven patients diagnosed with major depression and undergoing its maintenance treatment, and their primary family members were randomized to treatment as usual (TAU) only or to TAU plus family psychoeducation, which consisted of four 2-hour multiple-family sessions consisting of didactic lectures about depression (30 minutes) and group discussion and problem solving (60–90 minutes). The economic analyses were undertaken from the perspective of the National Health Insurance (NHI), assuming the most reasonable price of US$50 per psychoeducation session per patient. The main outcome measures included relapse-free days and direct costs to the NHI.

**Results:**

The intervention group enjoyed 272 (SD: 7.1) relapse-free days, while the control group spent 214 (SD: 90.8) relapse-free days (Cox proportional hazard ratio = 0.17, 95%CI: 0.04 to 0.75, p = 0.002). Cost-effectiveness acceptability curves suggested that the family psychoeducation has 90% or more chances of being cost-effective if the decision-maker is prepared to pay US$20 for one additional relapse-free day. This cost-effectiveness finding was robust when the price for family psychoeducation ranged between 50% to 150% of the baseline scenario in sensitivity analyses. If a relapse-free day is considered to be worth $30 or more, all the pricing scenarios have a close to 100% probability of being cost-effective.

**Conclusion:**

Family psychoeducation is effective in the relapse prevention of depression and is highly likely to be cost-effective if a relapse-free day is valued as US$20 or more.

**Trial registration:**

UMIN-CTR (UMIN000005555)

## Background

Depression is a prevalent [1], chronic [2] and relapsing [3] disorder. Pharmacotherapy, while moderately effective in alleviating acute depression [4] and in preventing relapse/recurrences [5], can nevertheless provide only partially satisfactory solutions to the patients’ sufferings [6]. It is evident that we need concerted efforts in both somatic and non-somatic approaches to lessen the disease burdens for these patients and their families. We have recently demonstrated the efficacy of a new approach to recurrent depression, namely family psychoeducation [7], which has been proven to be effective and cost-effective for the treatment of schizophrenia [8,9]. Whether family psychoeducation for depression is cost-effective, however, has never been examined.

This study presents an economic evaluation that we conducted alongside a randomized controlled trial to establish the cost-effectiveness of additional family psychoeducation versus no add-on treatment in the context of long-term continuation/maintenance treatment for major depression.

## Methods

### Participants

The details of the study participants and procedures are given elsewhere [7] and are briefly summarized below.

The patients were recruited at the Department of Psychiatry, Kochi Medical School**,** or its affiliated hospital, Doujin Hospital, between April 2004 and April 2006. The inclusion criteria for the patients were as follows:

1. Aged 18–85 years.

2. Diagnosed as suffering from major depressive disorder according to the DSM-IV[10]

3. Expected to be on continuation/maintenance antidepressant therapy for at least nine months after the patients had responded to acute phase antidepressant therapy and were in partial or full remission, i.e. they no longer fulfilled the diagnostic threshold for major depressive episode

4. Not having undergone electroconvulsive therapy or not having electroconvulsive therapy already planned for the index episode

5. 5. Living with the family for 3 months or longer before participating in this study and being expected to live with the family during the investigation period

6. Having at least one family member living with the patient who was available for family interviews.

The following patients were excluded:

1. Patients were screened with the Mini-Mental Status Examination [11] when dementia was clinically suspected and those scoring 23 or below were excluded.

2. Patients suspected of having organic diseases were examined using head magnetic resonance imaging, and those diagnosed as such were excluded.

The member of each family who was 18 years or older and who had contact with the patient for the longest time was regarded as his/her primary family member.

Of the 103 patients who met the above eligibility criteria, 57 patients and their family member provided written informed consent to participate in this study after full disclosure of the purposes and procedures of the study. The major reason for non-consent was that the primary family members were unable to participate in the four psychoeducation sessions because of their work.

This study was approved by the Ethics Committee of Kochi Medical School**,** and written informed consent was given by all the patients and their families. The trial is registered with UMIN-CTR (UMIN000005555).

### Procedure

The fifty-seven patients who consented were randomly allocated to the intervention and control groups and were followed up for nine months. A random sequence was generated using a random number table and was kept by an independent clerk who allocated the consecutive patient sample to the intervention or control groups. No stratification was used.

Both the intervention and control groups received the standard outpatient treatment, which was performed by psychiatrists who were kept unaware of the treatment allocation of the patients. This treatment as usual (TAU) consisted of an evaluation of psychiatric symptoms, assessment and management of drug treatment, and supportive psychotherapy on a bi-weekly basis. All the patients were kept on maintenance antidepressants.

The primary family members in the intervention group took part in family psychoeducation sessions with multiple primary family members without the participation of the patients. Only one family member per patient was allowed. The session was performed once every 2 weeks, and 4 sessions were regarded as one course. The themes of the four sessions were “Epidemiology and causes,” “Symptoms,” “Treatment and course,” and “Coping of the family with the patient.”

Each session lasted 90–120 minutes. The first 30 minutes were devoted to providing information regarding depression and its treatment. A video tape and a textbook explaining depression and its treatment were prepared for this study and were used as teaching materials. The next 60–90 minutes were devoted to group discussion and problem solving for emotionally difficult situations experienced by participating families.

In order to maximally facilitate group discussion, the number of participating family members was limited to five, while from the staff side**,** one leader and two co-leaders, usually consisting of two psychiatrists and one psychologist, attended each session. The whole program was supervised by SI, who had 30 years of experience in psychoeducation for people with severe mental illness. The sessions were videotaped, and the treatment team discussed their performance after the session was over.

### Cost and clinical assessments

The economic analyses were undertaken from the perspective of the direct costs to the National Health Insurance (NHI). In Japan**,** all medical expenditures are priced by the NHI, of which 70% are reimbursed by the NHI and 30% are paid by the patients out of their own pocket, except in special circumstances. We therefore asked the patients to collect all their NHI bills and summed them for the duration of the study. The sums included both the 70% reimbursed and the 30% paid out of the patients’ own pocket. Non-health service expenditures and indirect costs were not considered in the analyses. The costs in Japanese yen were all converted to US dollars at a rate of 100 yen/ 1 US dollar, which is the rough average exchange rate for the past several years at the time of writing of this manuscript (November 2011).

To evaluate the depressive state, we administered the Hamilton Rating Scale for Depression (HAM-D) [12] and the Beck Depression Inventory (BDI) [13] before intervention and after 9 months. The treating psychiatrist, who was blinded to the intervention status, administered the HAM-D. The BDI-II was filled in by the patient himself. When the treating psychiatrist who was blinded to the allocated intervention recognized the re-emergence of a major depressive episode according to the DSM-IV in the course of the bi-weekly visits constituting the treatment as usual, the patient was referred to an independent psychiatrist who also was kept blind to the intervention group and who administered the HAM-D and BDI. Relapse/recurrence was declared when the diagnostic threshold for a major depressive episode as specified in the DSM-IV was met according to the interview by this independent psychiatrist. The number of depression-free days up to the relapse/recurrence was taken as the unit of effectiveness.

### Analyses

The time to relapse/recurrence was compared between the two groups using a Cox proportional hazard analysis. The arithmetic means of the relapse-free days and the NHI costs were compared by way of the non-parametric bootstrap method because the data had highly skewed distributions [14].

Cost-effectiveness was evaluated by relating the differential cost per patient receiving either the intervention or the control treatment to the differential effectiveness of each treatment in terms of relapse-free days. The incremental cost-effectiveness ratio (ICER) was calculated as the difference in the mean cost divided by the difference in the number of relapse-free days.

A cost-effectiveness acceptability curve (CEAC) was drawn to show the probability that an intervention is cost-effective compared with the alternative, given the observed data, for a range of maximum monetary values that a decision-maker might be willing to pay for a particular unit change in outcome. The CEAC is derived from the joint distribution of incremental costs and incremental effects, as estimated by non-parametric bootstrapping of the observed data [15]. The range of maximum monetary values per relapse-free day is given on the x-axis. Given a specified value of this ‘acceptable’ cost-effectiveness ratio (a point on the x-axis), the CEAC shows the probability that the data are consistent with a true cost-effectiveness ratio falling below that value (read off the y-axis). This approach avoids the difficulties associated with the estimation of confidence intervals for the ICER.

In Japan, family psychoeducation is not yet covered by the NHI and therefore is not yet priced. In this RCT psychoeducation sessions were therefore administered as a research intervention without any cost to the NHI or the patients. In this cost-effectiveness analysis we estimated the most reasonable cost of family psychoeducation as follows and drew CEACs under this and two more cost scenarios whereby the price was estimated at 50% and 150% of the baseline scenario. Given that three therapists, whose average wage can range between US$30 to $50 per hour including social security costs, attended each 2-hour session for five patients, we calculated that the most reasonable estimate of one family psychoeducation session would be US$50 per patient. We also performed two sensitivity analyses in which a session would be priced at US$25 and $75 per patient.

All data were analyzed using SPSS 18.0. The bootstrap re-sampling was performed with STATA 11.0. Except where otherwise noted, the means of continuous variables are reported, followed by the SD in parentheses.

## Results

### Clinical outcomes

Of the 57 patient-family member pairs who originally consented and were randomized, 3 withdrew their consent after randomization (one family member in the intervention group and another in the control group refused to undergo the baseline assessments and one patient in the control group died from a physical illness), resulting in 24 and 30 evaluable patients for the intervention and control groups, respectively. The baseline characteristics of the two groups showed that the groups were closely comparable for all key variables (Table 1).

**Table 1:** **Baseline characteristics of the patients and the family members**

	**Intervention group (n = 24)**	**Control group (n = 30)**
Patients		
Sex (Male:Female)	15:9	15:15
Age (Year)	59.2 (14.6)	60.9 (13.0)
Illness duration (Year)	11.6 (2.7)	11.0 (2.0)
Number of previous admissions	0.8 (1.2)	0.8 (1.9)
Antidepressant dosage (imipramine equivalent)	100.3 (71.5)	88.1 (60.9)
HAM-D	13.4 (8.3)	13.7 (10.5)
BDI	12.4 (6.8)	12.0 (7.9)
Primary family members		
Father	2	0
Mother	0	3
Husband	7	13
Wife	14	12
Son	1	1
Daughter	0	1
Age(year)	59.0 (11.4)	61.8 (10.7)
Years of Education	12.0 (2.9)	10.7 (3.4)

HAM-D: Hamilton Rating Scale for Depression.

BDI: Beck Depression Inventory.

All 54 patients continued the treatment for 9 months and were assessed at follow-up. One patient in each group had stopped taking medication by the time of the follow-up. A relapse occurred before completion of the 9-month follow-up in 2 patients (8%) in the intervention group and in 15 patients (50%) in the control group (Cox proportional hazard ratio = 0.17, 95%CI: 0.04 to 0.75, p = 0.002). The intervention group enjoyed 272 (SD: 7.1, Range: 242 to 274) relapse-free days while the control group spent 214 (SD: 90.8, Range: 16 to 274) relapse-free days by the time of the 9-month follow-up (p = 0.009 using the bootstrap method).

### Cost outcomes

The total NHI costs were US$1,842 (SD: 2,656, Range: 173 to 9,600) for the intervention group and US$2,638 (SD: 5,605, Range: 197 to 24,472) for the control group (p = 0.509 using the bootstrap method). This increased cost was mainly due to a greater number of hospitalized days in the control group. Thus, the intervention was significantly superior to the control in terms of effectiveness and non-significantly so in terms of the total NHI cost when family psychoeducation was not charged.

However, if psychoeducation is properly priced and reimbursed, this cost difference may diminish or even be reversed. We therefore assumed the most reasonable price for a family psychoeducation session as well as two more cost scenarios whereby a session was priced at 50% and at 150% of this most reasonable estimate. Priced at US$50 session, the total NHI cost would rise to US$2,042; if priced 50% lower, it would rise to US$1942 and if priced 50% higher, it would rise to US$2142.

### Cost-effectiveness

We now examine the cost-effectiveness of the family psychoeducation under these costing scenarios when the additional benefits of increasing relapse-free days are taken into consideration.

**Figure 1:**
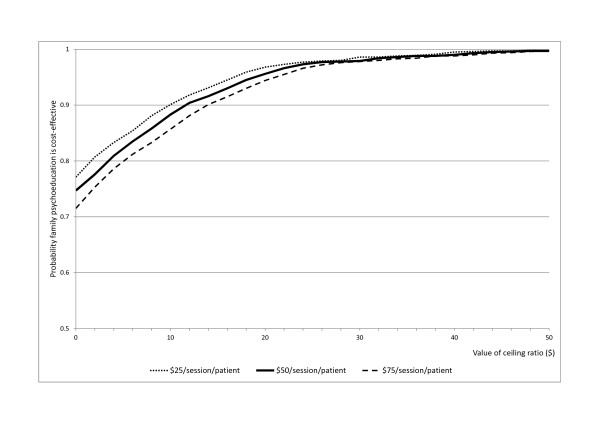
**Cost-effectiveness acceptability curves showing the probability that the treatment program is cost-effective in comparison with TAU (y-axis), as a function of a decision-maker’s ceiling cost-effectiveness value for one relapse-free day (x-axis), when the family psychoeducation session is priced at US$ 25, $50 or $75.**

Figure 1 presents the CEACs for family psychoeducation plus TAU over TAU alone under the three scenarios pricing attendance at a psychoeducation session at US$25, US$50 or US$75. The curve indicates the probability for the addition of family psychoeducation to be cost-effective for a range of potential maximum amounts (ceiling ratio) that a decision-maker is willing to pay for one more relapse-free day.

For example, even if the decision-maker does not value a relapse-free day at all, the family psychoeducation priced at US$50/session has an over 70% chance of being cost-effective. If the decision-maker is prepared to pay US$10 for one additional relapse-free day, the family psychoeducation priced at US$50/session has a close to 90% chance of being cost-effective. Sensitivity analyses indicate that even if the price of family psychoeducation is 50% greater and is priced at $75/session, it still has an over 80% chance of being cost-effective; the probability rises to 90% if priced at US$25/session.

If a relapse-free day is considered worth US$20, all the pricing scenarios considered in this study have an over 90% probability of being cost-effective. If a relapse-free day is considered worth US$30 or more, the probability of being cost-effective is nearly 100%.

## Discussion

We conducted a cost-effectiveness analysis of family psychoeducation in the context of a randomized controlled trial to compare its addition to TAU against TAU alone in the continuation/ maintenance treatment of major depression. Family psychoeducation significantly increased relapse-free days in comparison with TAU. The cost-effectiveness acceptability curves revealed that family psychoeducation is highly likely (>90%) to be cost-effective if one values one relapse-free day at or above US$20 to $30 when one session of family psychoeducation is priced at US$50 per patient. This cost-effectiveness finding was robust even when the price for a session ranged between 50-150% of the baseline scenario.

There are many ways to estimate the utility score for depression: one longitudinal study of patients receiving depression treatment concluded that an incremental quality-adjusted life year (QALY) from depressed to non-depressed state was 0.24 to 0.25 [16], while one cross-sectional study interviewing general population samples as well as formerly and currently depressed patients revealed that an incremental QALY between non-depressed state and moderate to severe depression ranged between 0.26 to 0.43 [17]. Because one QALY is often valued at US$50,000 to $70,000 [18], the monetary value of one depression-free day over one depressed day would then range between US$30 to $80. Although our relapse-free day may be valued somewhat less than a totally depression-free day, these figures would still place family psychoeducation at the rightmost end of the cost-effectiveness acceptability curves depicted in our Figure 1 and beyond. It must also be remembered that all the above arguments are based on direct heath care costs only. Indirect morbidity and mortality costs far outweigh the direct costs taken into consideration here [19,20].

Possible weaknesses of the current study may be as follows. First of all, the costing estimates were based on the prices as set by the Japanese NHI, and we cannot be sure how generalizable these estimates may be across different health care systems. Secondly, we were able to conduct sensitivity analyses around the psychoeducation cost only and were unable to conduct sensitivity analyses incorporating plausible ranges of drugs and other costs because our cost assessments did not include these figures separately. Thirdly, we were also unable to adopt a societal perspective to assess non-health care costs or indirect costs related to depression. Fourthly, the average age of the patients in this trial was around 60 years. The variability of our sample (2 in their 20s, 2 in their 30s, 6 in their 40s, 7 in their 50s, 9 in their 60s, 7 in their 70s, and one in their 80s) may be biased towards older generations, and its applicability to younger generations cannot be guaranteed, given the arguably different family dynamics at different ages. However, it must be emphasized that the effectiveness of the family psychoeducation remained statistically significant when age of the patient was entered into the Cox proportional hazard analysis [7]. Fifthly, the follow-up in the current study was only nine months. Whether the preventative effect of our four-session family psychoeducation lasts after this period is unknown. However, given the proportional hazard observed in the time to relapse analyses, such a possibility is more likely than not, and would further increase the cost-effectiveness of the intervention. On the other hand, even if the same relative decrease in relapse persists after 9 months, such benefits may have to be discounted, relative to more immediate gains as observed in the current study. Lastly, the family psychoeducation program that we experimented with in this trial may have particular strength in the Japanese context, where family ties are stronger than in other industrialized countries. The program may have to be slightly modified when transferred to other cultural settings, for example, by including the patients themselves if we want to extend the current program to more individualistic cultures.

## Conclusion

Despite these possible limitations, we believe that our economic analyses have provided compelling evidence that family psychoeducation in the maintenance treatment of depression is robustly cost-effective even when one extra relapse-free day is valued as low as being worth US$20 when family psychoeducation is priced at US$50 per session per person. The findings were robust when sensitivity analyses were conducted around this price estimate. It is hoped that, once the program and its (cost-)effectiveness are replicated in a few more trials in Japan and other countries, family psychoeducation may become an officially covered and widely provided practice in mental health services for people with depression and their families.

## Competing interests

In the last three years TAF has received honoraria for speaking at CME meetings sponsored by Astellas, Dainippon Sumitomo, Eli Lilly, GlaxoSmithKline, Janssen, Kyorin, Mochida, MSD, Meiji, Otsuka, Pfizer and Shionogi. He is on an advisory board for Sekisui Chemicals and the Takeda Science Foundation. He has received royalties from Igaku-Shoin, Seiwa-Shoten, Nihon Bunka Kagaku-sha and American Psychiatric Publication. The Japanese Ministry of Education, Science, and Technology and the Japanese Ministry of Health, Labor and Welfare have funded his research. All the other authors have no competing interests to declare.

## Author’s contributions

SS and KS conceived the study and wrote the protocol in consultation with YM, AT and SI. KS and SS ran the clinical trial on a day**-**to **-**day basis. TAF carried out the economic analyses and wrote the first draft of the manuscript. All the authors contributed to and have approved the final manuscript. SS is the guarantor.

## Funding

This study was supported by a Grand-in-Aid for Scientific Research, Ministry of Health, Labour and Welfare, 2004 (Comprehensive Research Project on Science of Longevity). The sponsor had no further role in the planning, conduct, analysis, writing or publishing of this study.

## Pre-publication history

The pre-publication history for this paper can be accessed here:

http://www.biomedcentral.com/1471-244X/12/40/prepub
